# Risk factors for ≥high-grade anal intraepithelial lesions in MSM living with HIV and the response to topical and surgical treatments

**DOI:** 10.1371/journal.pone.0245870

**Published:** 2021-02-03

**Authors:** Carmen Hidalgo-Tenorio, Carmen Maria García-Martínez, Juan Pasquau, Mohamed Omar-Mohamed-Balgahata, Miguel López-Ruz, Javier López-Hidalgo, Concepción Gil-Anguita

**Affiliations:** 1 Infectious Diseases Department, Virgen de las Nieves University Hospital, Granada, Spain; 2 Infectious Diseases Department, Hospital Complex of Jaen, Jaen, Spain; 3 Pathology Department, Virgen de las Nieves University Hospital, Granada, Spain; 4 Internal Medicine Department, Marina Baixa Hospital, Alicante, Spain; Azienda Ospedaliero Universitaria Ospedali Riuniti di Ancona Umberto I G M Lancisi G Salesi, ITALY

## Abstract

**Background:**

The objective of this study in MSM living with HIV was to determine the incidence of HSIL and ASCC, related factors, and the response to treatment.

**Patients and methods:**

Data were gathered in 405 consecutive HIV-infected MSM (May 2010-December 2018) at baseline and annually on: sexual behavior, anal cytology, and HPV PCR and/or high-resolution anoscopy results. They could choose mucosectomy with electric scalpel (from May 2010) or self-administration of 5% imiquimod 3 times weekly for 16 weeks (from November 2013). A multivariate logistic regression model was developed for ≥HSIL-related factors using a step-wise approach to select variables, with a significance level of 0.05 for entry and 0.10 for exit, applying the Hosmer-Lemeshow test to assess the goodness of fit.

**Results:**

The study included 405 patients with a mean age of 36.2 years; 56.7% had bachelor´s degree, and 52.8% were smokers. They had a mean of 1 (IQR 1–7) sexual partner in the previous 12 months, median time since HIV diagnosis of 2 years, and mean CD4 nadir of 367.9 cells/uL; 86.7% were receiving ART, the mean CD4 level was 689.6 cells/uL, mean CD4/CD8 ratio was 0.77, and 85.9% of patients were undetectable. Incidence rates were 30.86/1,000 patient-years for ≥high squamous intraepithelial lesion (HSIL) and 81.22/100,000 for anal squamous cell carcinoma (ASCC). The ≥HSIL incidence significantly decreased from 42.9% (9/21) in 2010 to 4.1% (10/254) in 2018 (p = 0.034). ≥HSIL risk factors were infection with HPV 11 (OR 3.81; 95%CI 1.76–8.24), HPV 16 (OR 2.69, 95%CI 1.22–5.99), HPV 18 (OR 2.73, 95%CI 1.01–7.36), HPV 53 (OR 2.97, 95%CI 1.002–8.79); HPV 61 (OR 11.88, 95%CI 3.67–38.53); HPV 68 (OR 2.44, CI 95% 1.03–5.8); low CD4 nadir (OR1.002; 95%CI 1–1.004) and history of AIDS (OR 2.373, CI 95% 1.009–5.577). Among HSIL-positive patients, the response rate was higher after imiquimod than after surgical excision (96.7% vs 73.3%, p = 0.009) and there were fewer re-treatments (2.7% vs 23.4%, p = 0.02) and adverse events (2.7% vs 100%, p = 0.046); none developed ASCC.

**Conclusions:**

HSIL screening and treatment programs reduce the incidence of HSIL, which is related to chronic HPV infection and poor immunological status. Self-administration of 5% imiquimod as first-line treatment of HSIL is more effective than surgery in HIV+ MSM.

## Introduction

Anal squamous-cell carcinoma (ASCC) is frequent among individuals living with HIV (HIV+) and associated with a high mortality rate [1] and with an incidence of up to 131/100,000 people/year among HIV+ men who have sex with men (MSM) [2], with a similar survival rate to the general population [3]. The beginning of a decline in its incidence among MSM has been described by some observational studies but not by clinical trials, which may be explained by the participation of their study populations in an intraepithelial neoplasia screening and treatment program [4, 5], as recently reported by our group [6].

ASCC has been associated with persistent infection by high-risk oncogenic HPV (HR-HPV) genotypes [7]. Interaction between HIV and HPV, which have risk factors in common, has been reported to increase the risk of HPV and reduce the clearance rate among individuals living with HIV [8].

It remains controversial whether the treatment of high-grade anal intraepithelial lesions (HSILs) prevents the subsequent development of ASCC [9], and no standard approach has been established, so that treatments vary among centers according to their resources and experience. Therapeutic options include local topical treatments with trichloroacetic acid, imiquimod, or 5-fluoruracil and more invasive treatments such as excision, fulguration, or laser therapy [9]. Surgical excision used to be a treatment of choice but is associated with adverse effects, including anal stenosis, and with recurrences and incomplete resection, especially when the anal disease is extensive [10]. Fulguration is applied during high-resolution anoscopy (HRA) and has been associated with a recurrence rate of around 25% at 30 months in HIV+ patients, depending on the amount of fulguration [11]. In regard to infrared or laser coagulation, it can be applied in the consulting room and is well tolerated, but a recurrence rate of 61% at one year has been described in HIV+ patients [12]. Finally, topical therapies offer a non-invasive option with a good safety profile and few adverse effects [9]. In particular, imiquimod offers the advantage of self-administration, a low recurrence rate, and a high response rate in HIV+ patients, which was reported to be 61% in a clinical trial [13].

In this study of consecutive HIV+ MSM undergoing a screening/treatment program at our hospital, we previously reported that oncogenic genotype clearance in anal mucosa was associated with the length of ART but did not affect the incidence of HSILs or ASCC. In the present article, data from the same population [6] were analyzed with a different objective, which was to determine the incidence of HSIL and ASCC, the risk factors, and the response to imiquimod treatment and/or electrosurgical excision.

## Patients and methods

### Design

This longitudinal observational (naturalistic/naturalist) prospective study was conducted in a population of 405 adult HIV+ MSM patients with no history of ASCC, who were consecutively enrolled between May 2010 and December 2018 in a screening and follow-up program for dysplasic anal mucosa lesions. This population and the methodology applied were previously reported [6] in a previous study on HR-HPV clearance and acquisition rates and their relationship with the incidence of HSILs/ASCC in anal mucosa. At their baseline visit (V0) we obtained the written informed consent of the patients to participation in the study, which was approved by the ethics committee (CEIC) of the University Hospital “Virgen de las Nieves” and complied with Spanish data protection legislation (Law 15/1999, 13 December). This CEIC is integrated within the network of ethics Committees of Andalusian public health system (SSPA). CEIC is regulated by order/Decree 439/2010 December 14th of Andalusia. All data were treated in accordance with Spanish data protection legislation Law 15/1999, 13 December, on Personal Character Data Protection).

In brief, data were gathered at baseline (V0), 4–12 weeks, and subsequent follow-ups (at individualized intervals) on clinical-epidemiological and analytical variables and on the results of PCR (*Gonorrhoeae*, *Mycoplasma spp*, *Chlamydia spp*, *Ureaplasma spp*) and oral-anal-urethral exudate culture (*Gonorrhoeae*) studies in symptomatic patients or infected partners. In addition, two anal canal mucosa samples were taken at baseline for HPV detection and genotyping by qualitative PCR and for cytology study using the “thin-layer” technique (Processor Thin Prep 2000 (Hologic), and HRA was performed at 4–12 weeks taking samples of apparently normal mucosa and areas with Lugol-negative aceto-white lesions using an endoscopic retrograde cholangiopancreatography catheter. HRA results were obtained from 100% of patients, and findings of at least one follow-up anoscopy were available for 97.3% (394/405). HRA was performed by an infectious disease specialist specifically trained for one year by an expert in this technique from the Department of Digestive Disease of our hospital.

At one year, patients with a normal anoscopy result and LSIL (AIN1) were examined with cytology, HPV PCR, and anoscopy, whereas those with HSIL underwent electrosurgical mucosal resection (from May 2010) or self-administration of 5% imiquimod 3 times/week for 16 weeks (from November 2013), followed by another anoscopy. Surgery was the sole option from May 2010 until the introduction of imiquimod in 2013; since then, all patients have been offered imiquimod as first treatment option. If the imiquimod treatment failed, patients were offered extension of the course up to 18 weeks, a new 16-week cycle of imiquimod, or surgery. Between May 2012 and May 2014, quadrivalent HPV vaccine was received by patients with no HPV 16 or 18 infection and no presence or history of HSIL+-compatible lesions. Cytology results were categorized according to the Bethesda classification [14] and histological findings according to the LAST HPV standardization project [15].

### Definition of variables

Abnormal cytology: Cytology findings of ASCUS, LSIL, or HSIL.

Histology with **≥**HSILs: Histology findings from HSIL to ASCC.

Therapeutic failure: HSIL persistence after treatment.

Post-treatment HSIL recurrence: Re-appearance of previously treated lesion with normal post-treatment anoscopy.

Response to treatment: Disappearance of lesion in follow-up anoscopy after treatment with surgery or imiquimod.

Self-administration of 5% imiquimod. On Mondays, Wednesdays and Fridays, patients used a preloaded single-use insulin syringe (after cleaning drug remains from its surface) to apply imiquimod inside the anal canal, 3 cm from the anal verge, while in left lateral decubitus position, preferably before sleeping. Before starting this therapy, the patient received detailed instructions on the procedure from the attending physician and nurse.

### Statistical analysis

SPSS 21.0 was used for data analyses. The descriptive statistics and tests used for bivariate analyses were previously reported in detail [6]. In the present study, a multivariate logistic regression model was developed for ≥HSIL-related factors, using Freeman’s formula [n = 10*(k+1)] [16], including significant variables in bivariate analysis (ART duration, infection with low-risk HPV genotypes, infection with HPV-6, 11, 16, 18, 53, 59, 61, or 68, and duration of infection with high-risk genotypes) and other variables deemed clinically relevant (smoking habit, history of AIDS, CD4 nadir, CD4/CD8 ratio, duration of infection with HR-HPV and mixed infection). A stepwise approach was used to select variables, with a significance level of 0.05 for entry and 0.10 for exit, applying the Hosmer-Lemeshow test to assess the goodness of fit. P≤0.05 was considered significant.

## Results

### Baseline characteristics of the cohort

Table 1 summarizes the baseline characteristics of the 405 HIV+MSM enrolled in the study, Table 2 exhibits the distribution of genotypes and Table 3 the cytology and anoscopy findings. In brief, the mean age was 36.2 years, and fewer than 10% were aged > 50 years. They had very good viro-immunological status, 85.6% were on ART at enrolment, and the mean interval since HIV diagnosis was around two years. There was a high prevalence of HPV infection: 76.9% with HR-genotypes, 73.1% with LR-genotypes and 58.1% with both. The prevalence of ≥HSILs was 21.7% (88/405), with an incidence of 30.86/1,000 patient-years, while the prevalence of ASCC was 0.74%, (4/405), with an incidence of 81.22/100,000 patient-years.

**Table 1:** Characteristics of HIV-infected MSM patients.

	Number of patients
	n = 405
**Age,** mean (± SD)	36.2 (± 10.1)
<30 yrs, n (%)	123 (30.4)
30–50 yrs, n (%)	244 (60.2)
>50 yrs n (%)	38 (9.4)
**Educational level**	
No studies	6 (1.5)
Primary studies	40 (9.9)
Secondary studies	129 (31.9)
University studies	230 (56.7)
**Retired, n (%), 95% CI**	22 (5.4) (3–7.5)
**Origin**	
Europe	387 (95.6)
Central America	17 (4.2)
**qHPV vaccine (2012–2014),** n (%), 95% CI	66 (16.3)
**Age at first sexual intercourse,** median (IQR)	18 (16–20)
**Number of lifetime male sex partners,** median (IQR)	50 (15–150)
**Number of male sex partners during previous 12 months,** median (IQR)	1(1–7)
**Habitual use of condoms,** n (%), 95% CI	294 (72.6) (68.2–77.4)
**Total number of sexual partners during follow-up,** median (IQR)	54.5(20–154)
**History of anal/genital warts,** n (%), (95%CI)	128 (31.6), (27.1–36.1)
**Anal/genital warts at baseline** n (%), (95%CI)	93 (23), (18.6–26.8)
**History of syphilis,** n (%), IC95%	103 (25.4), (21.6–29.8)
**History of other STI**, n (%), IC 95%	110 (27.2), (23.1–31.6)
**Time since HIV diagnosis (months),** median (IQR)	25 (8–84)
**CD4 at diagnosis of HIV (cel/uL),** mean (± SD)	448± 298.17
**HIV viral load at diagnosis of HIV (log),** median (IQR)	4.61 (4.07–5.12)
**CD4 nadir (cells/uL),** mean (± SD)	367.93±233.85
**CD4 nadir < 200 cells/uL,** n (%), 95% CI	97 (23.9), (20–28.5)
**CD4 cell count at baseline (cells/uL),** mean (± SD)	689.64± 475.03
**CD8 cell count at baseline (cells/uL),** mean (± SD)	981.5±531.5
**CD4 /CD8 ratio**, mean (± SD)	0.77±0.70
**HIV viral load at baseline (log),** median (IQR)	0 (0–1.72)
**Undetectable: < 50 HIV RNA copies/mL of plasma,** n (%)	348 (85.9)
**History of AIDS diagnosis,** n (%), 95% CI	106 (26.2) (21.3–30.1)
**HAART before inclusion,** n (%), 95% CI	351 (86.7), (83.2–90)
**Previous ART line,** median (IQR)	1 (1–2)
**Virological failure,** n (%)	17 (4.8)
**Median months of ART,** median (IQR)	4 (16–56)
**Chronic HCV infection,** n (%)	14 (3.5)
**Chronic HBV infection,** n (%)	13 (3.2)
**Smoker, pack/year,** median (IQR)	1.5 (0–14)
**Smoker,** n (%), 95% CI	214 (52.8) (47.9–57.4)
**Alcohol, SDU, median (IQR)**	0 (0–4)
**EX-IDU,** n (%)	2 (0.5)

HCV, **chronic infection by hepatitis C virus**; HBV, **chronic infection by hepatitis B virus;** EX-IDU**, ex-injecting drug users**; STI: **sexual transmitted infection;** VL**: viral load;** IQR**: interquartile range;** SD**, (standard deviation),** SDU**: standard drink unit.**

**Table 2:** Cytology, anoscopy, and HPV PCR results for the cohort.

	Cohort of MSM-HIV patients
	n = 405
**Anal cytology,** (n = 397), n (%), 95% CI	
LSIL	190 (47.9), (43–52.7)
HSIL	13 (3.3), (1.8–5.1)
ASCUS	29 (7.3), (5.1–10.2)
Normal	165 (41.6), (37–46)
**Anoscopy: Histology (**n = 405), n (%), 95% CI	
Normal	189 (46.7), (44.5–54)
LSIL	164 (40.4), (36.6–46)
HSIL	50 (12.3), (9.2–15.9)
ASCC	2 (0.5), (0–0.8)
**n (%), 95% CI**	**HPV PCR in anal mucosa**
	**n = 394**
HR-HPV	303 (76.9), (73–81.1)
LR-HPV	288 (73.1), (69–77)
HR and LR-HPV	229 (58.1), (53–63)
Median HR-HPV, IQR	1 (1–3)
Median LR-HPV, IQR	1 (0–2)
HPV 6	71 (18)
HPV 11	71 (18)
HPV 12	1 (0.3)
HPV 16	109 (27.7)
HPV 18	51 (12.9)
HPV 26	6 (1.5)
HPV 31	55 (14)
HPV 33	29 (7.4)
HPV 35	36 (9.1)
HPV 39	46 (11.7)
HPV-40	7 (1.8)
HPV-42	72 (18.3)
HPV-43	10 (2.5)
HPV 45	50 (12.7)
HPV 48	1 (0.3)
HPV 51	55 (14)
HPV 52	50 (12.7)
HPV 53	36 (9.1)
HPV 54	26 (6.6)
HPV 55	64 (16.2)
HPV 56	31 (7.9)
HPV 58	23 (5.8)
HPV 59	42 (10.7)
HPV 61	30 (7.6)
HPV 62	56 (14.2)
HPV 64	1 (0.3)
HPV 66	34 (8.6)
HPV 68	42 (10.7)
HPV 69	14 (3.6)
HPV 70	32 (8.1)
HPV 71	1 (0.3)
HPV 72	28 (7.1)
HPV 73	37 (9.4)
HPV 81	51 (12.9)
HPV 82	17 (4.3)
HPV 83	5 (1.3)
HPV 84	30 (7.6)
HPV 89	1 (0.3)
HPV 6108	13 (3.3)
HPV-AR subtype of HPV 18 (39,45,59,68)	164 (41.6)
HPV-AR subtype of HPV 16 (31,33,35,52,58,67)	207 (52.5)

LSIL, **low-grade squamous intraepithelial lesion**; HSIL, **high-grade squamous intraepithelial lesion**; ASC, **atypical squamous cells of undetermined significance**; ASCC, **anal squamous cell cancer**. HPV, **human papillomavirus;** HR-HPV**: high-risk HPV,** LR-HPV**: low-risk HPV.**

**Table 3:** Risk factors associated with ≥HSILs in HIV+ MSM patients. Bivariate and multivariate analysis.

	≥HSIL	NORMAL	Bivariate	Multivariate
	N = 88	N = 317	p*	OR 95% CI
**Mean age (yrs), mean** (± DS)	30.4 (± 7.6)	31.3 (± 8.3)	0.359	1.27 (0.71–2.29)
**Retired, n (%)**	6 (6.8)	16 (5)	0.594	
Smoker, n (%)	50 (56.8)	164 (51.7)	0.398	
Charlson Index, median (IQR)	0 (0–0)	0 (0–0)	0.178	
**Intercourse in previous 12 months, n (%)**	78 (88.6)	286 (90.8)	0.545	
**qHPV Vaccine, n (%)**	**1**2 (13.6)	54(17)	0.445	1.72 (0.92–3.23)
**Age at first sexual intercourse, (IQR)**	18 (17–21)	18(16–20)		
**Genital/anal warts, n (%)**	36(40.9)	92(29)	0.034	
**History of Syphilis, n (%)**	24(27.3)	79(24.9)	0.654	
**HCV infection, n (%)**	4 (4.5)	10 (3.2)	0.515	
**HBV infection, n (%)**	2 (2.3)	11 (3.5)	0.742	
**Total NPS, baseline visit,** median, (IQR)	50 (16–200)	50 (19.5–150)	0.543	
**NSP12m before last visit**, median, (IQR)	1 (1–4)	1 (1–6.5)	0.078	
**Use of condom during study,** n (%)	63 (71.6)	244 (76.9)	0.493	
**History of AIDS (A3, B3, C),** n (%)	29 (33)	77(24.3)	0.102	**2.37(1.009–5.58)**
**Time since HIV diagnosis** (months), (IQR)	27 (9–83)	54(21–107)	0.386	1.002 (1.000–1.003)
**CD4 nadir (cells/ul), mean (± SD)**	366.8(±267.9)	368.2(±223.9)	0.959	
**CD4 nadir < 200 cells/uL, n (%)**	24(27.6)	73(23.3)	0.412	
**Cd4 nadir <500 cells/uL, n (%)**	39 (44.8)	161 (51.4)	0.275	
**Cd4 nadir >500 cells/uL, n (%)**	24 (27.6)	77 (24.6)	0.571	
C**D4 cells/uL, mean (± SD)**	674.3(± 347.3)	761.2(± 406.9)	0.069	0.99(0.99–1)
**CD8 cells/uL, mean (± SD)**	1017.3(± 474.1)	977.5(± 467.5)	0.487	
**CD4/CD8, mean (± SD)**	0.75(± 0.43)	0.85(± 0.41)	0.042	0.53(0.2–1.38)
**HIV VL (log), mean (± SD)**	4.09 (± 3.35)	4.91(± 4.27)	0.042	1 (1–1)
**ART during follow-up, n (%)**	78 (88.6)	300 (94.6)	0.046	0.74 (0.22–2.56)
**Median months of ART, median, (IQR)**	0 (0–13)	24 (0–48)	0.003	0.99(0.99–1)
**Virological failure, n (%)**	1 (1.3)	4 (1.3)	1	
	**N = 83**	**N = 291**		
**Infection by Low-risk HPV genotype, n (%)**	65 (78.3)	211 (72)	**0.349**	
**Infection by High-risk HPV genotype, n (%)**	68 (83.9)	174 (59.8)	**0.0001**	2.72(0.87–8.54)
**Infection by Low and High-risk HPV, n (%)**	53 (63.9)	132 (45.4)	**0.003**	0.79 (0.25–2.46)
**N° of HR-HPV genotypes, median (IQR)**	2 (1–3)	1 (0–2)	**0.0001**	0.76 (0.58–1)
**N° of LR-HPV genotypes, median (IQR)**	1 (1–2)	1 (0–2)	**0.082**	0.83 (0.61–1.12)
**Median months with VPH-AR (IQR)**	11 (1–18)	1 (0–24)	**0.07**	0.98 (0.96–1.02)
**Median months with VPH-BR (IQR)**	8 (1–16)	11 (0–26)	**0.487**	1 (0.96–1.04)
**Median months with mixed VPH infection (IQR)**	1 (0–15)	1 (0–12)	**0.102**	
**HPV-6**	21 (25.3)	47 (16.2)	0.057	1.61 (0.73–3.53)
**HPV-11**	21 (25.3)	37 (12.7)	0.005	**3.81(1.76–8.24)**
**HPV-16**	27 (32.5)	43 (14.8)	0.0001	**2.69 (1.22–5.99)**
**HPV-18**	16 (19.3)	26 (8.9)	0.008	**2.73 (1.01–7.36)**
**HPV-31**	10 (12)	29 (10)	0.584	
**HPV-33**	4 (4.9)	17 (5.9)	0.733	
**HPV-35**	6 (7.2)	18 (6.1)	0.781	
**HPV-39**	9 (10.8)	24 (8.2)	0.462	
**HPV-42**	12 (14.5)	53 (18.3)	0.419	
**HPV-45**	9 (10.8)	31 (10.7)	0.961	
**HPV-51**	12 (14.5)	26 (8.9)	0.142	
**HPV-52**	10 (12)	40 (13.7)	0.689	
**HPV-53**	12 (14.5)	13 (4.5)	0.001	**2.97 (1.002–8.79)**
**HPV-54**	6 (7.2)	24 (8.2)	0.763	
**HPV-55**	11 (13.3)	50 (17.2)	0.393	
**HPV-59**	11 (13.3)	19 (6.5)	0.047	1.8(0.66–4.83)
**HPV-61**	11 (13.3)	8 (2.7)	0.001	**11.88 (3.67–38.53)**
**HPV-68**	16 (19.3)	32 (11)	0.048	**2.44(1.03–5.8)**
**HPV-70**	8 (9.8)	24 (8.2)	0.667	
**HPV-81**	10 (12)	56 (19.2)	0.129	

P*: p-value

95% CI: 95% confidence interval

HIV+MSM, **men who have sex with men living with HIV;** LTI, **Latent tuberculosis infection;** HCV **hepatitis C virus**; HBV, **hepatitis B virus**; HPV, **Human papillomavirus**; EX-IDU, **ex-injecting drug addict**; VL, **viral load.** HR-HPV**: high-risk HPV,** LR-HPV**: low-risk HPV;** LSIL, **low-grade squamous intraepithelial lesion;** HSIL, **high-grade squamous intraepithelial lesion;** ASC, **atypical squamous cells of undetermined significance, NSPt, Total number of sexual partners; NSP12m: number of sexual partners in past 12 months**

### Factors associated with ≥HSIL

In the multivariate analysis, the presence of ≥HSILs was related to infection with HPV genotypes 11 (OR 3.81; CI95% 1.76–8.24), 16 (OR 2.69, 95%CI 1.22–5.99), 18 (OR 2.73, CI95% 1.01–7.36), 53 (OR 2.97, CI95% 1.002–8.79), 61 (OR 11.88, 95%CI 3.67–38.53) and 68 (OR 2.44, 95%CI 1.03–5.8), low CD4 nadir (OR1.002; CI95% 1–1.004), and history of AIDS (OR 2.37, 95%CI 1.009–5.58). The remaining results obtained are exhibited in Table 3.

### HSIL patients and treatment outcomes

After excluding 11 patients who did not undergo two or more anoscopies, treatment outcomes were analyzed in a sample of 394 (87.2%) MSM, with a median follow-up of 36 months (IQR: 12–69), 1.215 patients-year. Table 4 exhibits the characteristic of the two HSIL treatment groups (imiquimod and surgery).

**Table 4:** Baseline characteristics of HIV+MSM patients receiving imiquimod vs. surgery.

	Imiquimod as first option	Surgery as first option	Bivariate
	N = 32	N = 47	p*
**Mean age (yrs), mean (± DS)**	35.3.4 (± 11.48)	31.3 (± 8.3)	0.88
**Retired, n (%)**	2 (6.3)	3 (6.4)	1
**Smoker, n (%)**	19 (59.4)	29 (61.7)	0.68
**Intercourse in previous 12 months, n(%)**	28 (87.5)	47 (100)	0.72
**qHPV Vaccine, n (%)**	6 (18.7)	6 (12.8)	0.36
**Age at first sexual intercourse, (IQR)**	18 (16–21)	18(16–20)	0.53
**Genital/anal warts, n (%)**	12 (37.5)	23 (48.9)	0.53
**History of Syphilis, n (%)**	11(34.3)	13 (27.7)	0.33
**HCV infection, n (%)**	1 (3.1)	3 (6.4)	1
**HBV infection, n (%)**	0 (0)	2 (4.3)	0.53
**Total NPS, baseline visit, median, (IQR)**	55 (30–300)	36 (15–200)	0.14
**NSP12m before last visit, median, (IQR)**	2 (1–9)	1 (1–8)	0.55
**Use of condom during study, n (%)**	26 (81.3)	39 (82.9)	0.51
**History of AIDS (A3, B3, C), n (%)**	11 (34.4)	15 (31.9)	0.56
**Time since HIV diagnosis (months), (IQR)**	21 (7–111)	25 (9.5–64.3)	0.92
**CD4 nadir (cells/ul), mean (± SD)**	366.8(±267.9)	368.2(±223.9)	0.96
**CD4 nadir < 200 cells/uL, n (%)**	6(18.8)	15(31.9)	0.36
**Cd4 nadir 200–500 cells/uL, n (%)**	15 (46.9)	24 (51.1)	0.78
**Cd4 nadir >500 cells/uL, n (%)**	10 (31.3)	14 (29.8)	0.57
**CD4 cells/uL, mean (± SD)**	618.2(± 280.3)	675.7(± 334.1)	0.42
**CD8 cells/uL, mean (± SD)**	946.4(± 443.9)	1096(± 551.5)	0.17
**CD4/CD8, mean (± SD)**	0.78(± 0.46)	0.70(± 0.35)	0.42
**HIV VL (log), mean (± SD)**	3.9 (± 4.53)	3.55(± 3.98)	0.42
**ART during follow-up, n (%)**	28 (87.5)	45 (95.7)	0.6
**Median months of ART, median, (IQR)**	19.5 (6.5–44)	9 (2–64)	0.57
**VL HIV < 50 copies/uL, n (%)**	20(62.5)	39 (82.9)	0.28
**Infection by Low-risk HPV genotype, n (%)**	32 (100)	41 (87.2)	0.29
**Infection by High-risk HPV genotype, n (%)**	26 (81.3)	46 (97.8)	0.52
**Infection by Low and High-risk HPV, n (%)**	18 (56.3)	34 (72.3)	0.40
**Sub-species HPV 18 (18, 39, 45, 59, 68)**	16 (50)	29 (61.7)	0.61
**Sub-species HPV 16 (16, 31, 33, 35, 52, 58, 67)**	17 (53.1)	23 (48.9)	0.43
**N° of HR-HPV genotypes, median (IQR)**	2 (1–3.8)	2 (1–3)	0.28
**N° of LR-HPV genotypes, median (IQR)**	2 (0.3–3)	1 (1–2)	0.92

P*: p-value

95% CI: 95% confidence interval

HIV+MSM, **men who have sex with men living with HIV;** LTI, **Latent tuberculosis infection;** HCV **hepatitis C virus**; HBV, **hepatitis B virus**; HPV, **Human papillomavirus**; VL, **viral load.** HR-HPV**: high-risk HPV,** LR-HPV**: low-risk HPV;** LSIL, **low-grade squamous intraepithelial lesion;** HSIL, **high-grade squamous intraepithelial lesion;** ASC, **atypical squamous cells of undetermined significance, NSPt, Total number of sexual partners; NSP12m: number of sexual partners in past 12 months.**

Mucosectomy was performed in 47 patients with HSIL, with a median follow-up of 60 months (IQR: 46–73 months) and median disease-free period of 48 months (IQR: 28–60 months).

Forty-one (87.2%) of these patients received surgery alone, five (10.6%) received surgery plus self-administered imiquimod, due to failure of surgery in two cases and recurrence in three, and one patient underwent successful surgery after the failure of imiquimod treatment. Surgical margins were disease-free in 35 patients (76.9%) after first surgery. The response rate was 73.3% to surgery as first-line treatment (33/45) *versus* 96.8% to imiquimod (31/32) (p = 0.009) as evaluated by post-treatment HRA. A median of one surgical intervention was performed (IQR:1–1), with 31 patients undergoing one, 9 needing two, and 1 needing four interventions. Recurrence was recorded in 7 patients (15.2%), and repeat excision of the same lesion in 11 (23.4%). All surgical patients reported adverse effects, with a median duration of 15 days post-surgery (IQR: 7–21 days), including bleeding with defecation in 32 (68%), pain requiring anti-analgesics in 39 (82.9%), rectal incontinence in 1 (2.1%), and transient anal stenosis in 3 (6.4%) *versus* 1 in the imiquimod group (2.7%, p = 0.046). Among the 43 patients with follow-up HPV PCR results, clearance of oncogenic VPH genotypes was observed in 19 (44.2%).

Thirty-seven patients with HSIL self-administered 5% imiquimod three times/week; the treatment lasted 16 weeks in 97.3% of these patients and 18 weeks in 2.7%. It was first-line treatment in 32 patients (86.4%) and administered after previous surgery in 5 (13.5%); all patients showed a complete response, except for one case of failure caused by intolerance to imiquimod. Only one patient (2.7%) needed retreatment of the same lesion, whereas 11 (23.4%) of the surgical group required repeat surgery (p = 0.02). The median number of affected quadrants was 1 (IQR: 1–2). Mean follow-up was 48 months (IQR: 35–57 months) and mean disease-free period 36 months (IQR: 12–48). Imiquimod was discontinued in one patient (2.7%) for adverse effects (anal itching, stinging, and/or pain) attributed to non-compliance with the treatment protocol. Among the 35 (94.6%) patients treated with imiquimod for whom follow-up HPV PCR results were available, clearance of oncogenic VPH genotypes was observed in 10 (28.6%) (p = 0.065). No patient treated with surgery or imiquimod progressed to ASCC.

We found significant reductions in ≥HSIL cases between 2010 and 2018 (42.9% (9/21) *vs*. 4.1% (10/245) p = 0.034), between 2010 and 2013 (42.9% (9/21) *vs*. 13.8% (22/159), p = 0.003), and between 2013 and 2016 (13.8% (22/159) *vs*. 4.8% (13/273), p = 0.0001), followed by a stabilization between 2016 and 2018 (4.8% (13/273) *vs*. 4.1% (10/245), p = 0.617). Four deaths were recorded during the follow-up: one patient with hepatic cirrhosis secondary to chronic HCV infection in 2012, one with Burkitt lymphoma in 2013, one with small-cell lung cancer in 2014, and one with metastatic ASCC in 2015. Further data on outcomes were previously reported in detail [6].

## Discussion

Since the first HIV epidemic, the incidence of ASCC has increased in seropositive patients, mainly in MSM with AIDS [17]. After the initiation of our screening/treatment program in 2010, the ≥HSIL rate significantly decreased among HIV-infected MSM patients for six years and then subsequently stabilized, with no progression to ASCC in patients with treated HSIL. Our results support the proposition by the authors of the Swiss Cohort study that the incidence of ASCC among people living with HIV can be markedly reduced if they all receive ART and can be further diminished if they also undergo annual screening with anal cytology or anoscopy [18]. A recent study of 592 HIV patients, with a mean follow-up of 69 months, reported that the risk of progression from HSIL (AIN3) to ASCC was high and that ASCC screening was the only factor that reduced this risk [19]. Data from the Study for the Prevention of Anal Cancer (SPANC) [20] are expected to elucidate the natural evolution of HPV infection, allowing a more effective classification of patients at risk of ASCC. In the meantime, a program to screen, diagnose, treat and follow up anal mucosal dysplastic lesions appears recommendable, especially in HIV+ MSM.

In this prospective study of HIV+ MSM undergoing a screening/treatment program for anal mucosa dysplasic lesions, the presence of ≥high-grade anal intraepithelial lesions were related to infection by HPV genotypes 11 16, 18, 53, 61 and 68, a low CD4 nadir and a history of AIDS. This finding of a relationship between HSIL-positivity and poor immunological status is consistent with previous observations that prolonged antiretroviral treatment [21–23] and a high CD4 count, regardless of CD4 nadir [24], are protective factors against HSIL. A recent prospective study observed a similar incidence of HPV-16 and -18 genotypes in the anal mucosa of French HIV+MSM, but HPV-16 was more persistent and therefore more closely correlated with the presence of HSIL [25]. In a retrospective study of “alpha-human papillomavirus” in the anal mucosa of German HIV+ patients, the presence of HSIL and simultaneous infection was associated with high- and low-risk genotypes [26]. Currently, patients with a new diagnosis of HIV in Spain are usually MSM, and the diagnosis is late in 47.6% of these cases [27], with a CD4 count <200 cells/uL. The above data suggest that screening for anal dysplasia is essential in this type of patient.

Self-administration of 5% imiquimod was a highly effective therapeutic strategy against HSIL in this series of HIV+MSM. Most of them did not need to repeat the topical treatment, whereas around a quarter of the patients undergoing excision required another intervention. Furthermore, the therapeutic failure rate and dropout for adverse effects were lower in the imiquimod group than in the surgery group. Various studies have supported the efficacy of imiquimod to treat HSIL in HIV+ patients [13, 28, 29]. Thus, a double-blind randomized placebo-controlled clinical trial comparing between self-application of imiquimod (n = 28) *versus* placebo (n = 25) in the anal canal three times/week for 4 months found a significant association (P = 0.003) between imiquimod and a positive outcome [13]. In addition, a prospective, observational open study in 44 HIV+ patients with HSIL observed a response rate of 66% (29/44) for imiquimod [28]. Finally, a retrospective observational study in 28 HIV+ and HIV- patients observed a higher frequency of total or partial responses in those receiving anal tampon treatment with a 15 mg *versus* 6.25 mg dose of imiquimod, with no difference in CD4, HIV viral load, or serostatus [29].

Further advantages of 5% imiquimod in comparison to ablative therapies include its self-administration and its usefulness in cases of extensive disease. The surgical option was also effective in a large proportion of our patients, although some needed retreatment due to recurrence or incomplete excision. No cases of permanent stenosis or fecal incontinence were observed in the surgical group; however, surgery is not currently recommended due to its adverse effects, especially in patients with large lesions [30].

Limitations of this single-center study include its observational design, comparing the real-life clinical effectiveness of imiquimod and surgery rather than their efficacy (as in a clinical trial). In addition, it only included HIV+MSM, and these data cannot be extrapolated to other types of patient. Finally, 11 of the 405 enrolled patients did not undergo two or more anoscopies and were therefore lost to the follow-up. However, its strengths include the prospective design and long follow-up period, which was a mean of 36 months. In fact, the present cohort of HIV+ patients is one of the few published to date that was created to measure predetermined objectives.

In conclusion, HSIL screening and treatment programs reduce the incidence of this precursor of ASCC. Chronic mixed HPV infection and a history of poor immunological status are associated with the presence of HSILs. Self-administration of 5% imiquimod is more effective than surgery as first-line treatment of anal HSIL in HIV+MSM patients, with a lower recurrence rate and fewer adverse effects.

## Supporting information

S1 File HIV MSM cohort database. (SAV)
